# A Search for Blood Biomarkers for Autism: Peptoids

**DOI:** 10.1038/srep19164

**Published:** 2016-01-14

**Authors:** Sayed Zaman, Umar Yazdani, Yan Deng, Wenhao Li, Bharathi S. Gadad, Linda Hynan, David Karp, Nichole Roatch, Claire Schutte, C. Nathan Marti, Laura Hewitson, Dwight C. German

**Affiliations:** 1 Department of Psychiatry, UT Southwestern Medical Center, Dallas TX.; 2 Department of Clinical Sciences, UT Southwestern Medical Center, Dallas TX.; 3 Department of Internal Medicine, UT Southwestern Medical Center, Dallas TX.; 4 Johnson Center for Child Health and Development, Austin TX.; 5 University of Texas, Austin TX.

## Abstract

Autism spectrum disorder (ASD) is a neurodevelopmental disorder characterized by impairments in social interaction and communication, and restricted, repetitive patterns of behavior. In order to identify individuals with ASD and initiate interventions at the earliest possible age, biomarkers for the disorder are desirable. Research findings have identified widespread changes in the immune system in children with autism, at both systemic and cellular levels. In an attempt to find candidate antibody biomarkers for ASD, highly complex libraries of peptoids (oligo-N-substituted glycines) were screened for compounds that preferentially bind IgG from boys with ASD over typically developing (TD) boys. Unexpectedly, many peptoids were identified that preferentially bound IgG from TD boys. One of these peptoids was studied further and found to bind significantly higher levels (>2-fold) of the IgG1 subtype in serum from TD boys (n = 60) compared to ASD boys (n = 74), as well as compared to older adult males (n = 53). Together these data suggest that ASD boys have reduced levels (>50%) of an IgG1 antibody, which resembles the level found normally with advanced age. In this discovery study, the ASD1 peptoid was 66% accurate in predicting ASD.

Autism spectrum disorder (ASD) is a neurodevelopmental disorder characterized by impaired social communication and interaction, and restricted, repetitive patterns of behavior^1^. Approximately 1 in 70 children are diagnosed with ASD at an average age of 4 years, according to the CDC’s Autism and Developmental Disabilities Monitoring Network^2^. Early therapeutic intervention has been found to lessen the burden of ASD to the children and their families^3^,^4^. A blood-based biomarker for ASD would facilitate early intervention with behavioral therapies. Such a biomarker approach has been undertaken recently by several investigators^5^,^6^.

The immune system has been linked with ASD^7^. Abnormalities in both serum antibody concentrations and T cells have been reported for ASD compared to typically developing (TD) children^8^,^9^,^10^. Immunological anomalies in children with ASD include altered cytokine profiles^11^,^12^,^13^, decreased immunoglobulin levels^14^, altered cellular immunity^15^, and neuroinflammation^16^. Autoimmunity has also been described for autism with several studies reporting circulating autoantibodies to neural antigens^17^,^18^.

The present study sought to examine the immune system to search for antibodies in the blood that may be related to ASD. We employed an approach previously used to search for antibody biomarkers for Alzheimer’s disease^19^, neuromyelitis optica^20^, and systemic lupus erythematosus^21^, using libraries of synthetic compounds containing oligomers of N-substituted glycines, called peptoids. These peptoid libraries can be readily synthesized to contain millions of unique compounds, putatively covering a vast range of chemical space^22^. With this approach, the peptoid compounds can serve to mimic naturally occurring epitopes and can be used to screen for antibodies in an unbiased fashion. We describe here our finding of a peptoid identified by this method that can discriminate between ASD and TD male serum based upon its ability to bind antibody.

## Results

### Identification and validation of “hit” peptoids

**Figure 1:**
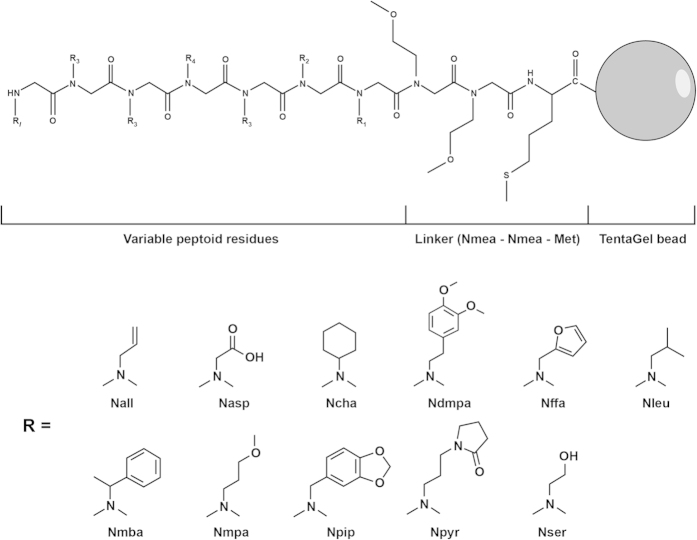
Configuration of the first library used to screen for ASD-related compounds. Abbreviations: Met = methionine; Nall = allylamine; Nasp = glycine; Ncha = cyclohexylamine; Nffa = furfurylamine; Nleu = isobutylamine; Nmba = (R)-methylbenzylamine; Nmea = 2-methoxyethylamine; Nmpa = 3-methoxypropylamine; Nphe = benzylamine; Npip = piperonylamine; Npyr = N-(3′-aminopropyl)-2-pyrrolidinone; Nser = ethanolamine.

**Figure 2:**
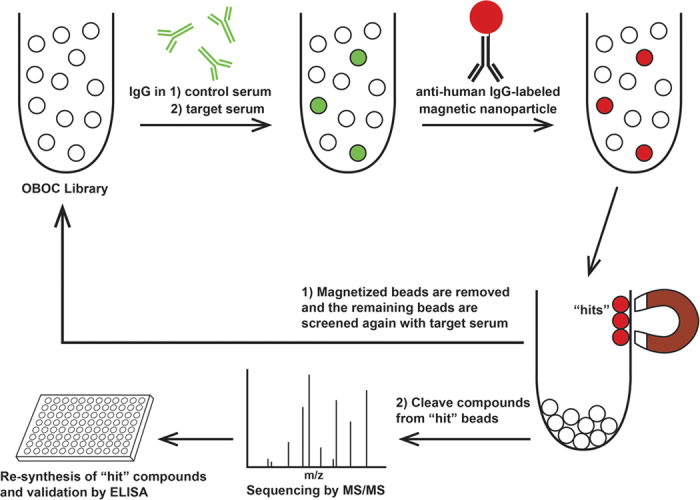
On-bead magnetic screening. A one-bead one-compound (OBOC) library of thousands of unique peptoid compounds bound to TentaGel beads is incubated with control serum, here serum pooled from TD subjects. The library is then incubated with anti-human IgG-labeled magnetic nanoparticles so that beads having bound IgG from the serum can be sorted out using a strong magnet. The library is initially depleted of beads that bind IgG from the control serum, and then incubated with target serum, here serum pooled from ASD subjects. After incubation with the magnetic nanoparticles again, the newly magnetized beads, called “hits”, are isolated. Peptoid compounds are cleaved from each of the “hit” beads and their sequences are assessed by MS/MS. These “hit” compounds are then re-synthesized and validated on ELISA plates for their ability to detect target IgG.

In an effort to isolate peptoid compounds that bind antibodies specific to children with ASD versus TD, three distinct one-bead one-compound peptoid libraries were synthesized and screened using serum pools from both groups. The first library consisted of 10-mer compounds with 7 variable peptoid residues that could be any of 10 different amines, yielding a theoretical diversity of 10^7^ possible compounds (Fig. 1). During screening, the library was first depleted of peptoids that bound IgG in serum pooled from 10 TD males. The depleted library was then incubated with serum pooled from 10 ASD males. Compounds that were then found to bind IgG were designated as “hit” peptoids (Fig. 2). A second library was synthesized in an attempt to reduce the large number of nonspecific “hit” beads yielded during screens with the first library. This library was designed to be less hydrophobic in character by the inclusion of the charged residue, Nlys (diaminobutane), in both the invariant linker and as a possible amine in the variable region. Finally, a third library was synthesized with a theoretical diversity lower than the previous two — only 200,000 possible compounds — to encourage the isolation of redundant compounds during screening as an intermediate measure for validating the specificity of “hits”^23^. Screens of these latter libraries were performed with the same serum pools in the same way as with the first.

Using the same two serum pools as used during screening, the “hit” peptoids were then tested by ELISA for their ability to bind IgG from the two pools. Based on the screening design, and as observed in previous studies with other sample sets^20^,^21^,^24^, it was expected that successful validation of a “hit” peptoid would demonstrate the compound to bind *higher levels* of IgG from the ASD pool than from the TD pool. In total, 25 “hit” peptoids were isolated from all three libraries, identified, re-synthesized, and tested in this way by ELISA. None of them, however, satisfied the expectation for a successful validation. Rather, all of them, independent of the library from which they were isolated, uniformly demonstrated the opposite pattern of binding in which they bound higher levels of IgG from the TD pool than from the ASD pool.

### Assessment of ASD1

**Figure 3:**
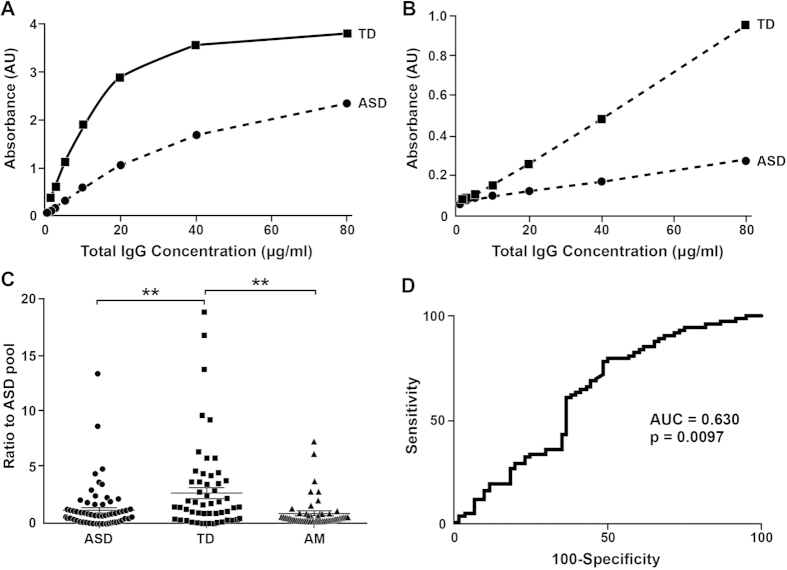
Serum IgG binding to the ASD1 peptoid. **(A)** Titration of IgG binding to ASD1 using serum pooled from 10 TD males and 10 ASD males demonstrates ASD1’s ability to differentiate between the two groups. **(B)** Detecting IgG1 subclass instead of total IgG amplifies this differentiation. **(C)** IgG1 binding of individual ASD (n=74) and TD (n=60) male serum samples (1:100 dilution) to ASD1 significantly differs with TD>ASD. In addition, IgG1 binding of older adult male (AM) serum samples (n=53) to ASD1 is significantly lower than TD males, and not different from ASD males. The three groups were compared with a Kruskal-Wallis ANOVA, H = 10.1781, p<0.006. **p<0.005. Error bars show SEM. **(D)** Receiver-operating characteristic curve for ASD1’s ability to discriminate between ASD and TD males.

Although the 25 compounds isolated from the various screens did not demonstrate the ability to bind antibodies elevated in ASD, they were able to consistently differentiate between the ASD and TD serum pools in levels of IgG binding. A peptoid called ASD1, isolated from the first library, was chosen to further assess the nature of this differentiation (Fig. 3). Figure 3, panels A,B show how the differentiation in binding between the serum pools can be amplified from two-fold to nearly four-fold using a secondary antibody specific to the IgG1 subclass. Using this same secondary antibody, serum samples from individual ASD (n = 74) and TD (n = 60) male subjects were tested against ASD1 (Fig. 3C). Consistent with the serum pool results, ASD1 binding of IgG1 from TD males (2.65 ± 0.50; mean ± SEM) was significantly higher than that of IgG1 from ASD males (1.17 ± 0.23; Mann Whitney; p = 0.009). The receiver-operating characteristic curve for discriminating ASD vs. TD was 0.630, p = 0.0097 (Fig. 3D). The ASD1 peptoid had an Accuracy of 66%, a Sensitivity of 78% and a Specificity of 51% in predicting ASD vs. TD.

We also compared binding of the ASD1 peptoid to serum samples from older adult males of mean age 66.7 years (n = 53). ASD1 binding of IgG1 from the older adult males did not differ from that of the ASD males, but was significantly lower than that of the TD males (Mann Whitney; p = 0.004) (Fig. 3C).

We examined ASD1 binding to a small sample of females; the binding to ASD (n = 10) and TD (n = 20) females was 1.76 ± 0.81 and 1.97 ± 0.38, respectively which is intermediate to the values found in ASD and TD males. The ASD1 levels was similar in the TD females and the TD males, and there was no significant decrease in ASD1 binding in the ASD female vs. TD subjects (both males and females) (Kruskal-Wallis H = 0.54, ns).

### Association between peptoid binding and ADOS and ADI-R subdomains

Higher scores in any domain on the ADOS and ADI-R are indicative of more abnormal behaviors and/or symptoms. Among ADOS subdomains, there was no significant relationship between Communication and peptoid binding (*z* = 0.04, *p* = 0.966), Communication + Social interaction (*z* = 1.53, *p* = 0.127), or Stereotyped Behaviors and Restrictive Interests (SBRI) (*z* = 0.46, *p* = 0.647). Higher scores on the Social Interaction domain were significantly associated with higher peptoid binding (z = 2.04, *p* = 0.041).

Among ADI-R subdomains, higher scores on the Communication domain were associated with lower levels of peptoid binding (*z* = −2.28, *p* = 0.023). There was not a significant relationship between Social Interaction (z = 0.07, *p* = 0.941) or Restrictive/Repetitive Stereotyped Behaviors (z = −1.40, *p* = 0.162) and peptoid binding.

### Isolation of ASD1-binding proteins

**Figure 4:**
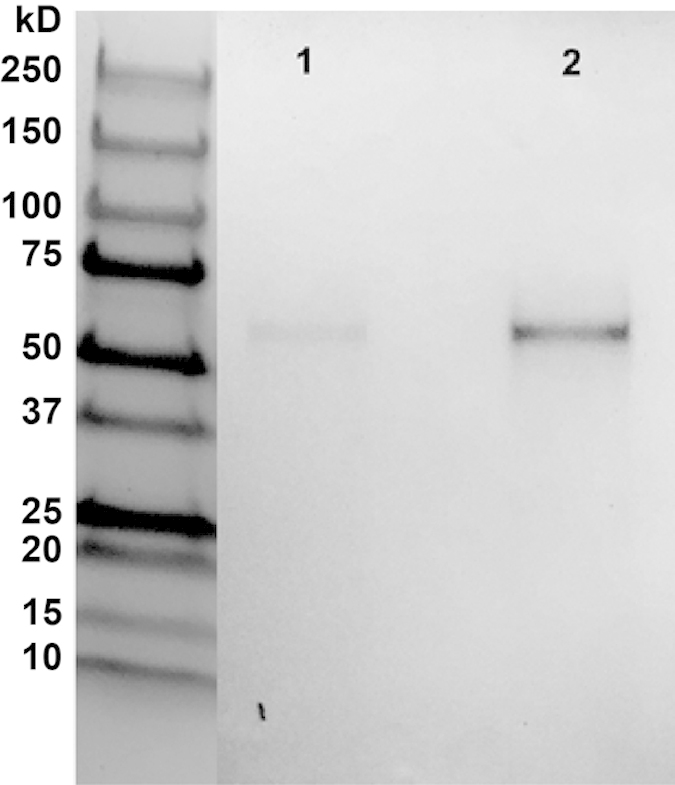
Assessment of proteins that bind to ASD1. ASD1 peptoid was immobilized and incubated with pooled serum from ASD or TD males. Serum was removed and what proteins were left bound to ASD1 were eluted out and evaluated by gel electrophoresis and Coomassie Blue staining. Lane 1 shows ASD1 pull-down analytes from the ASD serum pool and Lane 2 shows the pull-down from the TD serum pool. Both show a single band at ~55–60 kD that is higher in intensity for the TD male analyte.

In an attempt to identify the antibody or antibodies recognized by ASD1, affinity purification with ASD1 was performed against the same ASD and TD serum pools used during screening. The proteins left bound to ASD1 were eluted out and analyzed by gel electrophoresis and Coomassie Blue staining. A single band was observed whose molecular weight is ~55–60 kD (Fig. 4). The band is observable in both ASD and TD analytes, and its intensity is higher in the TD analyte, which correlates with the IgG1 binding to ASD1 observed with ELISA.

## Discussion

Peptoid libraries have been used previously to search for autoantibodies for neurodegenerative diseases^19^ and for systemic lupus erythematosus (SLE)^21^. In the case of SLE, peptoids were identified that could identify subjects with the disease and related syndromes with moderate sensitivity (70%) and excellent specificity (97.5%). Peptoids were used to measure IgG levels from both healthy subjects and SLE patients. Binding to the SLE-peptoid was significantly higher in SLE patients vs. healthy controls. The IgG bound to the SLE-peptoid was found to react with *several* autoantigens, suggesting that the peptoids are capable of interacting with multiple, structurally similar molecules. These data indicate that IgG binding to peptoids can identify subjects with high levels of pathogenic *autoantibodies* vs. a single antibody.

In the present study, the ASD1 peptoid binds significantly *lower levels* of IgG1 in ASD males vs. TD males. This finding suggests that the ASD1 peptoid recognizes antibody(-ies) of an IgG1 subtype that is (are) significantly lower in abundance in the ASD males vs. TD males. Although a previous study^14^ has demonstrated lower levels of plasma IgG in ASD vs. TD children, here, we additionally quantified serum IgG levels in our individuals and found no difference in IgG between the two groups (data not shown). Furthermore, our IgG levels did not correlate with ASD1 binding levels, indicating that ASD1 does not bind IgG generically, and that the peptoid’s ability to differentiate between ASD and TD males is related to a specific antibody(-ies).

ASD subjects underwent a diagnostic evaluation using the ADOS and ADI-R, and application of the DSM-IV criteria prior to study inclusion. Only those subjects with a diagnosis of Autistic Disorder were included in the study. The ADOS is a semi-structured observation of a child’s behavior that allows examiners to observe the three core domains of ASD symptoms: reciprocal social interaction, communication, and restricted and repetitive behaviors^1^. When ADOS subdomain scores were compared with peptoid binding, the only significant relationship was with Social Interaction. However, the positive correlation would suggest that lower peptoid binding is associated with better social interaction, not poorer social interaction as anticipated.

The ADI-R is a structured parental interview that measures the core features of ASD symptoms in the areas of reciprocal social interaction, communication and language, and patterns of behavior. Of the three ADI-R subdomains, only the Communication domain was related to ASD1 peptoid binding, and this correlation was negative suggesting that low peptoid binding is associated with greater communication problems. These latter data are similar to the findings of Heuer *et al.*^14^ who found that children with autism with low levels of plasma IgG have high scores on the Aberrant Behavior Checklist (p < 0.0001). Thus, peptoid binding to IgG1 may be useful as a severity marker for ASD allowing for further characterization of individuals, but further research is needed.

It is interesting that in serum samples from older men, the ASD1 binding is similar to that in the ASD boys. This is consistent with the observation that with aging there is a reduction in the strength of the immune system, and the changes are gender-specific^25^. Recent studies using parabiosis^26^, in which blood from young mice reverse age-related impairments in cognitive function and synaptic plasticity in old mice, reveal that blood constituents from young subjects may contain important substances for maintaining neuronal functions. Work is in progress to identify the antibody/antibodies that are differentially binding to the ASD1 peptoid, which appear as a single band on the electrophoresis gel (Fig. 4).

There are some limitations regarding this study. Since recruitment for the current study was limited to ASD subjects with a diagnosis of autistic disorder, none of whom were taking psychiatric medications, we could control for some potential confounding factors. The increased prevalence of ASD in males resulted in the study group including more males than females, which does not allow one to thoroughly investigate any gender-specific differences. However, the data from this study suggest that even with the relatively small sample size, gender-specific differences appear to be present. This observation should be further evaluated in a larger study.

Blood biomarkers for diseases have become a topic of great recent interest. Such biomarkers are relatively non-invasive for the patient and inexpensive to analyze. Panels of biomarkers have been used to identify Alzheimer’s disease (AD); panels consisting of 10–20 proteins have been identified which allow ~90% Sensitivity and Specificity for the identification of AD^27^, and panels of plasma phospholipids have proven useful for the identification of AD^28^, and even panels of pathogenic proteins (i.e., amyloid β1-42 and P-T181 tau)^29^. For ASD, biomarker searches are relatively new. A study using metabolomics^5^ identified a panel of >100 mass features to produce an optimal predictive signature for ASD in a small sample ASD and TD children. The Accuracy of prediction for this panel was 81%. More recently, a blood-based panel of genomic biomarkers was identified for young boys with ASD^6^. The Accuracy of this panel was 75%. This signature of differentially expressed genes was enriched in translation and immune/inflammation functions. When we combined ASD1 binding with thyroid stimulating hormone levels measured in the same 43 ASD and 37 TD boys (data submitted for publication) we find a predictive Accuracy of 73% as opposed to 66% for the ASD1 peptoid alone. Because *panels of blood proteins* can provide a high Sensitivity/Specificity for identifying a disease, we are studying whether the ASD1 peptoid, in combination with inflammatory blood analytes, can serve as a useful *biomarker panel* for identifying ASD.

## Methods

### Human subjects

The study protocol and all subsequent amendments were submitted by The Johnson Center for Child Health and Development (Austin, TX) and approved by the Austin Multi-Institutional Review Board (AMIRB) for ASD and TD children, and the Institutional Review Board at UT Southwestern (UTSW) Medical School for adults. The study was carried out in accordance with these approved protocols.

The ASD group was comprised of 74 male subjects with a median age of 5.6 years (range 2.3–9.5 years). The TD group was comprised of 60 males with a median age of 6.3 years (range 2.5–8.9 years). We also used 10 ASD females (median age of 5.3; range: 2.8–6.7 years) and 20 TD females (median age of 5.4, range: 2.2–7.5 years). These subjects were either recruited directly from The Johnson Center, or through the use of informational study flyers. Written informed consent was received from the parent or guardian of all subjects prior to enrollment. For the ASD group, all subjects were assessed by The Johnson Center clinical psychologist using both the Autism Diagnostic Observation Schedule (ADOS) and the Autism Diagnostic Interview–Revised (ADI-R). Clinical diagnosis was made based on these data and overall clinical impression using DSM-IV criteria. For this particular study, subjects with a diagnosis of Asperger’s Syndrome or Pervasive Developmental Disorder - Not Otherwise Specified (PDD-NOS), were excluded. Only those subjects meeting the criteria for Autistic Disorder were included. For the TD group, all subjects underwent a developmental screening using the Adaptive Behavior Assessment System - Second Edition (ABAS-II). TD subjects were excluded if their score on the ABAS-II suggested possible concerns for development and the need for further evaluation. TD subjects were also excluded if they had any first- or second-degree relatives diagnosed with ASD. Subjects diagnosed with a genetic, metabolic, or other concurrent physical, mental, or neurological disorder were also excluded, as were any subjects that were currently taking psychiatric medications (or had taken psychiatric medications within the last 3 months prior to enrollment).

Normal control older adult male serum samples (n = 53) were obtained from the UT Southwestern Medical School’s Alzheimer’s Disease Center and the Parkinson’s Disease Biomarker Program. All subjects were cognitively normal and free from neurodegenerative diseases based upon clinical evaluation, neuropsychological testing and in some cases brain scans. The median age of these subjects was 69 years (range 40–75 years).

### Blood collection and storage

A fasting blood draw was performed on ASD and TD subjects between the hours of 8–10 a.m. Blood was collected into a 3.5 ml Serum Separation Tube (SST; Vacutainer System; Becton-Dickinson) using standard venipuncture technique. The blood was gently mixed in the SST by 5 inversions and then stored upright for clotting at room temperature for 30 mins. Blood was spun immediately after the clotting time in a swing bucket rotor for 20 minutes at 1100-1300 × g at room temperature. Serum was removed immediately after centrifugation and transferred into coded cryovials in 0.25 ml aliquots. Aliquots of serum were immediately placed upright in specimen storage box in a −20 °C freezer for up to 6 hours. Samples were then transferred to a −80 °C freezer for long-term storage. Sample aliquots were shipped to UTSW on dry ice. The blood from adult male control subjects was collected and stored according to protocols established by the Alzheimer’s Disease Neuroimaging Initiative (see http://www.adni-info.org/Scientist/Pdfs/adni_protocol 9 19 08.pdf).

### Peptoid library synthesis

Three distinct one-bead one-compound combinatorial libraries of peptoids (oligo-N-substituted glycines) were synthesized onto 75 μm TentaGel beads using a split and pool method^22^. Library 1 was configured as NH_2_-X_7_-Nmea-Nmea-Met-TentaGel, where X = Nall, Nasp, Ncha, Nffa, Nleu, Nmba, Nmpa, Nphe, Npip, or Nser, yielding a theoretical diversity of 10^7^ possible compounds (Fig. 1). (Monomer abbreviations: Met = methionine, Nall = allyamine, Nasp = glycine, Nbsa = 4-(2-aminoethyl) benzenesulfonamide, Ncha = cyclohexylamine, Ndmpa = 3,4-dimethoxyphenethylamine, Nffa = furfurylamine, Nippa = 3-isopropoxypropylamine, Nleu = isobutylamine, Nlys = 1,4-diaminobutane, Nmba = (R)-methylbenzylamine, Nmea = 2-methoxyethylamine, Nmpa = 3-methoxypropylamine, Nphe = benzylamine, Npip = piperonylamine, Npyr = N-(3′-aminopropyl)-2-pyrrolidinone, Nser = ethanolamine). Library 2 was configured as NH_2_-X_6_-Nmpa-Nlys-Met-TentaGel, where X = Nall, Nasp, Ncha, Nippa, Nleu, Nlys, Nmba, Npip, Npyr, Nser (theoretical diversity = 10^6^ possible compounds). Library 3 was configured as NH_2_-X_5_-Nmea/Nlys-Ndmpa-Nmea-Met-TentaGel, where X = Nall, Nasp, Nbsa, Nippa, Nleu, Nlys, Nmba, Npip, Npyr, Nser (theoretical diversity = 200,000 possible compounds). Methionine linkers were coupled in the usual way, while the peptoid residues were coupled using the submonomer method^30^ with microwave irradiation to accelerate reactions^31^. Proper library syntheses were confirmed by CNBr cleavage of compounds from samples of isolated beads and subsequent analysis by tandem mass spectrometry.

### On-bead magnetic screening

A modification of the magnetic capture method for screening on-bead libraries^24^ was used. Approximately 375,000 beads from the library were soaked in PBST (PBS-0.1% Tween 20, pH 7.4) and then blocked with blocking buffer (1:1 mixture of 1% bovine serum albumin (BSA) in PBST and SuperBlock Blocking Buffer (Thermo Scientific, Rockford, IL)) for 1 hr. at room temperature (RT). Serum aliquots from 10 TD males were pooled and then diluted up to 1 ml in blocking buffer to obtain a final IgG concentration of 40 μg/ml. IgG levels of serum pools were measured using Human IgG ELISA Quantitation Set (Bethyl Laboratories, Montgomery, TX). The library beads were then incubated with the diluted serum in a cryovial overnight at 4 °C. The beads were washed with PBST eight times and re-suspended in blocking buffer. A 10 mg/ml anti-human IgG-conjugated Dynabead solution, 50 μl, was prepared by coupling 10 μg of biotinylated Goat F(ab)_2_ anti-human IgG (Southern Biotech, Birmingham, AL) with 0.5 mg of Dynabead M-280 Streptavidin (Invitrogen, Grand Island, NY). The library beads were mixed with the Dynabead solution for 2 hrs. at room temperature (RT) with gentle agitation. Library beads with high levels of bound Dynabeads were then separated by placing the tube in a strong magnetic field. These “magnetized” beads were removed from the library. The remaining beads were again washed and the magnetic capture was repeated two more times, completing the depletion. The depleted library was then incubated with pooled serum aliquots from 10 ASD males as described above. “Hit” beads were obtained by performing the magnetic capture and collecting the magnetized beads. “Hit” peptoid compounds were then identified by CNBr cleavage of compounds from “hit” beads and sequencing by MALDI TOF/TOF (Fig. 2).

For validation and subsequent analyses, “hit” peptoid compounds were re-synthesized on Polystyrene AM RAM resin (Rapp Polymer, Tübingen, Germany) with the methionine linker, as in the library, replaced by a cysteine linker so that the compounds could be immobilized using sulfhydryl-reactive chemistry. Peptoid compounds were cleaved off the resin by incubating in a 95% trifluoroacetic acid, 2.5% triethylsilane, 2.5% water solution for 2 hrs. at RT. Peptoid compounds were subsequently purified using high-performance liquid chromatography and verified by MS analysis.

### Peptoid ELISA

Peptoid compounds were immobilized onto maleimide-activated 96-well plates (Pierce Biotechnology, Rockford, IL) by dissolving them to 0.03–0.05 mM in a 0.1 M sodium phosphate, 0.15 M sodium chloride, 10 mM EDTA solution adjusted to pH 7.2 and incubating with shaking for 3 hrs. at RT. Plates were washed with PBST and blocked with a 5% goat serum (Thermo Scientific, Rockford, IL) in PBST solution for 1 hr. at RT. Plates were washed again and incubated with target (serum) samples diluted in blocking buffer (1:1 PBST-1%BSA and SuperBlock) for 2 hrs. at RT. After washing, plates were incubated with Goat anti-Human IgG-Fc-HRP conjugate (Bethyl Laboratories, Montgomery, TX) diluted 1:30,000 in PBST-1%BSA *or* mouse anti-human IgG1 (hinge)-HRP conjugate (SouthernBiotech, Birmingham, Alabama) diluted 1:1000 in PBST-1%BSA for 30 min at RT. After another wash, plates were incubated in TMB substrate for 16 min at RT and stopped with 2 M H_2_SO_4_. Plates were read at 450 nm. All samples were run in duplicate, and every assay contained ASD and TD serum pool samples to serve as internal controls. Results for individual samples were assessed as ratios to the ASD serum pool so as to control for plate-to-plate variation.

For control experiments, total IgG levels for individual serum samples were quantified using human IgG ELISA Quantitation Set (Bethyl Laboratories, Montgomery, TX), and IgG1 levels were quantified using IgG Subclass Human ELISA Kit (Invitrogen, Grand Island, NY).

### Affinity purification of peptoid-binding proteins

The ASD1 peptoid was coupled to iodoacetyl gel columns from MicroLink Peptide Coupling Kit (Thermo Scientific, Rockford, IL) at concentrations of 3–5 mM. Regarding the step requiring blocking of excess iodoacetyl groups with l-cysteine, identical protein bands were observed in preliminary gels with or without the inclusion of this step. Thereafter, the step was omitted in subsequent iterations. ASD1-coupled gel columns were then incubated with 1:60 dilutions of serum in PBST for 2 hrs. at RT with gentle agitation. Flow-through was discarded and peptoid-binding proteins were collected by elution with 100 μl of the low-pH buffer provided. We used 5 μl of 1 M Tris, pH 9.0 added to each eluted aliquot to neutralize the low pH. The eluted aliquots were then each lyophilized and re-dissolved in 10 μl of PBS to maximize the protein concentration for visualization on the gel.

### Gel electrophoresis and Coomassie Blue staining

Peptoid-binding protein analytes were loaded into 4–20% Mini-Protean TGX Precast gels (Bio-Rad Laboratories, Hercules, CA) after mixing with 5 μl 10% 2-mercaptoethanol in Laemmli Sample Buffer (Bio-Rad Laboratories, Hercules, CA) and heating at 95 °C for 5 min. After electrophoresis, the gel was stained with 0.1% Coomassie Blue R-250 (Bio-Rad Laboratories, Hercules, CA) in a 10% acetic acid, 50% methanol, 40% water solvent for 2 hrs. at RT with gentle agitation. Gels were then destained overnight at RT in the same solvent with gentle agitation and two changes of solvent throughout.

### Statistics

Regression analyses were conducted using the R lavaan package, which fits models using full information maximum likelihood estimation using all available data. Thus, data from all participants was included in each model^32^. All other statistical analyses were performed using GraphPad Prism 6. The mean values of un-transformed ELISA data for individual samples were compared by Kruskal-Wallis H-test, and Mann-Whitney U-tests. Peptoid binding was regressed on each of the ADOS and ADI-R subdomain scores to examine whether ASD1 peptoid binding was related to ‘clinically relevant quantitative traits’ of ASD. Prior to fitting regression models, peptoid binding was log-transformed to reduce the positive skew; the transformed distribution was approximately normally distributed and met guidelines for covariance matrix based models^33^. ROC and cutscore methods were used to determine the accuracy of the ASD1 peptoid for predicting ASD vs. TD. Medians and range were used to describe the 2 groups of ASD and TD boys and the Mann-Whitney U test was used to compare groups on ASD1 levels. To explore the use of a cut score for group prediction, ROC analysis was used. A variety of criteria were used to define the optimal cut score for these data; (1) the maximum perpendicular distance from (and above) the 45 degree line of equality^34^, (2) highest accuracy (correct predictions), and (3) best sensitivity/specificity combination. Chi^2^ test of independence was used to examine the predictions using the new cut score. SPSS V23 was used to perform the statistical analyses.

## Additional Information

**How to cite this article**: Zaman, S. *et al.* A Search for Blood Biomarkers for Autism: Peptoids. *Sci. Rep.* **6**, 19164; doi: 10.1038/srep19164 (2016).
